# High Efficiency of Temperate *Aedes albopictus* to Transmit Chikungunya and Dengue Viruses in the Southeast of France

**DOI:** 10.1371/journal.pone.0059716

**Published:** 2013-03-18

**Authors:** Anubis Vega-Rua, Karima Zouache, Valerie Caro, Laure Diancourt, Pascal Delaunay, Marc Grandadam, Anna-Bella Failloux

**Affiliations:** 1 Department of Virology, Institut Pasteur, Arboviruses and Insect Vectors, Paris, France; 2 Department of Infection and Epidemiology, Institut Pasteur, Genotyping of Pathogens and Public Health, Paris, France; 3 Hôpital de l’Archet, Centre Hospitalier Universitaire de Nice, and Inserm U1065/Université de Nice-Sophia Antipolis, Laboratoire de Parasitologie–Mycologie, Nice, France; 4 Department of Virology, Institut Pasteur, Molecular Interactions Flavivirus-Hosts, National Reference Center for Arboviruses, Paris, France; Centro de Pesquisas René Rachou, Brazil

## Abstract

**Background:**

Since 2005, cases of chikungunya (CHIK) were caused by an unusual vector, *Aedes albopictu*s. This mosquito, present in Europe since 1979, has gained importance since its involvement in the first CHIK outbreak in Italy in 2007. The species is capable of transmitting experimentally 26 arboviruses. However, the vectorial status of its temperate populations has remained little investigated. In 2010, autochthonous cases of CHIK and dengue (DEN) were reported in southeastern France. We evaluated the potential of a French population of *Ae. albopictus* in the transmission of both viruses.

**Methodology and Principal Findings:**

We used two strains of each virus, CHIK and DEN: one strain was isolated from an imported case, and one from an autochthonous case. We used as controls *Aedes aegypti* from India and Martinique, the source of the imported cases of CHIK and DEN, respectively. We showed that *Ae. albopictus* from Cagnes-sur-Mer (AL-CSM) was as efficient as the typical tropical vector *Ae. aegypti* from India to experimentally transmit both CHIK strains isolated from patients in Fréjus, with around 35–67% of mosquitoes delivering up to 14 viral particles at day 3 post-infection (pi). The unexpected finding came from the high efficiency of AL-CSM to transmit both strains of DENV-1 isolated from patients in Nice. Almost 67% of *Ae. albopictus* AL-CSM which have ensured viral dissemination were able to transmit at day 9 pi when less than 21% of the typical DEN vector *Ae. aegypti* from Martinique could achieve transmission.

**Conclusions/Significance:**

Temperate *Ae. albopictus* behaves differently compared to its counterpart from tropical regions, where recurrent epidemic outbreaks occur. Its potential responsibility for outbreaks in Europe should not be minimized.

## Introduction

In September 2010, a few cases of dengue (DEN) and chikungunya (CHIK) were reported in the southeast of France [1], [2]. Patients did not move outside France during the preceding weeks, suggesting an autochthonous transmission of both viruses by the mosquito *Aedes albopictus*. This species, native to Southeast Asia, has expanded all over the world during the past decades, including to the south of Europe (reviewed in [3]). Since its first report in Albania in 1979 [4], *Ae. albopictus* has been found in 20 European countries including France [5]. This mosquito was first detected in 2004 in the city of Menton near the Italian border, and has gained ground each year. It is now established in seven French departments [6].

*Ae. albopictus* is able to transmit a large number of arboviruses (reviewed in [7]). Laboratory viral challenges gave evidence of the high efficiency of *Ae. albopictus* from Europe (Corsica and Italy) to ensure dissemination of CHIK virus (CHIKV) and to a lesser extent, DEN virus (DENV) [8], [9]. However, its vectorial status in temperate regions has been debatable for a long time until the first European epidemic of CHIK in 2007 in Italy [10]. Its implication as vector has been corroborated in 2010 with the detection of CHIK and DEN cases in France and Croatia [1], [2], [11]–[13]. These unexpected cases illustrate the risk for European countries of arboviral outbreaks, supported by increasing numbers of viraemic travelers returning from endemic tropical regions [14].

In September 2010, two autochthonous cases of DENV-serotype 1 (DENV-1) were reported in Nice (southeastern France). Molecular genotyping revealed that DENV-1 strains were similar to strains circulating in Martinique in 2010 [1]. At the same time, 50 km west of Nice, in Fréjus, two autochthonous cases of CHIK were detected [11]. Viral genome sequences were identical to that isolated from a traveler returning from India (Rajasthan) [2]. A local transmission of both viruses has then been suspected as it coincided with the occurrence of high densities of *Ae. albopictus* [3], [5]. The main aim of our study is to assess whether the temperate counterpart of *Ae. albopictus* collected in southeastern France shows a high ability to transmit CHIKV and DENV when compared to *Ae. aegypti*, the typical vector of both viruses in tropical endemic regions.

## Materials and Methods

### Ethics Statement

The Institut Pasteur animal facility has received accreditation from the French Ministry of Agriculture to perform experiments on live mice [see permit numbers at http://webcampus.pasteur.fr/jcms/c_97619/agrements-des-animaleries] in appliance of the French and European regulations on care and protection of the Laboratory Animals. This study was approved by the Institutional Animal Care and Use Committee (IACUC) at the Institut Pasteur. No specific permits were required for the described field studies in locations which are not protected in any way and did not involve endangered or protected species.

### Mosquitoes

Three colonies of *Aedes* mosquitoes were used: (i) *Ae. albopictus* collected in 2007 in the city of Cagnes-sur-Mer (AL-CSM) in southeast France; (ii) *Ae. aegypti* collected in 2010 in the district “Le Robert” in the French overseas department of Martinique (AA-Martinique); and (iii) *Ae. aegypti* from New-Delhi in India (AA-India). All colonies were derived from field-collected eggs. After hatching, larvae were split by 200 individuals per pan, fed with 1 yeast tablet dissolved in 1 liter of tap water. Emerging adults were maintained in cages at 28°C±1°C with a light:dark cycle of 16 h:8 h, 80% relative humidity, and were supplied with a 10% sucrose solution. The F13 and F2 generations were respectively used for AL-CSM and AA-Martinique. AA-India has been maintained in laboratory since 1985.

### Viral Strains

Four viral strains isolated from patients in Southeastern France were used: CHIKV (2010–1630 and 2010–1909) and DENV-1 (2010–1806 and 2010–2025). CHIKV 2010–1630 and DENV 2010–2025 were isolated from imported cases returning respectively from India and Martinique, whereas CHIKV 2010–1909 and DENV 2010–1806 were obtained from autochthonous cases who have not traveled during the preceding months. Viral strains were produced on African green monkey kidney Vero cells. The 2^nd^ passage was used for experimental infections of mosquitoes. Serial dilutions were used to determine the titer of viral stocks that was expressed in plaque-forming units (PFU)/mL for CHIKV and focus-forming units (FFU)/mL for DENV.

### Viral Genome Sequencing and Phylogenetic Analyses

Viral genomic RNA was extracted from CHIKV grown once on mosquito C6/36 cells. RT-PCR was performed using SuperScript One-Step RT-PCR with platinum Taq (Invitrogen) using primers targeting the complete genome [15]. Amplicons sequencing reactions were performed by using Big Dye Terminator v1.1 cycle sequencing kit (Applied Biosystems) and sequencing was performed in ABI3730XL sequence analyzer (Applied Biosystems). For sequence analysis, contig assembly and sequence alignments were performed using program BioNumerics version 6.5 (Applied-Maths, Sint-Martens-Latem, Belgium). For phylogenetic analysis, maximum-likelihood tree was constructed using MEGA version 5 (www.megasoftware.net), based on the Tamura-Nei model. Reliability of nodes was assessed by bootstrap resampling with 1,000 replicates.

### Mosquito Oral Infections

One-week-old females were fed on an infectious blood-meal containing 2 mL of washed rabbit erythrocytes, 1 mL of viral suspension supplemented with a phagostimulant (ATP) at a final concentration of 5 mM. The titer of the blood-meal was determined at 10^7.3^ PFU/mL for CHIKV and 10^5.3^ FFU/mL for DENV. Fully engorged females were transferred in cardboard containers and maintained with 10% sucrose at 28±1°C.

### Dissemination and Transmission Analysis

Batches of mosquitoes were analyzed at days 3, 6, 9 and 14 post-infection (pi) for CHIKV [15] and at days 9 and 14 pi for DENV [16].

To estimate the dissemination rate, heads were removed from mosquitoes and ground in 500 µL of Leibovitz L15 medium supplemented with 10% Fetal Bovine Serum (FBS). Obtained homogenates were then filtered with a Millipore® membrane (0.22 µm) for further titration. To estimate transmission, saliva was collected from individual mosquitoes as described in [17]. For each mosquito, wings and legs were removed and the proboscis was inserted into a 20 µL tip containing 5 µL of FBS. After 45 min, FBS containing saliva was expelled in 45 µL of Leibovitz L15 medium (Invitrogen) for titration.

Disseminated infection rate corresponds to the proportion of mosquitoes with infected head among tested ones. Transmission rate represents the proportion of mosquitoes with infectious saliva among mosquitoes able to disseminate the virus beyond the midgut barrier. Ultimately, transmission efficiency corresponds to the proportion of mosquitoes with virus in saliva among tested ones (i.e., surviving females including females unable to disseminate the virus and those able to disseminate).

### Viral Titration of Mosquitoes

The number of infectious particles per saliva and per head homogenates was estimated by focus fluorescent assay on C6/36 *Ae. albopictus* cells. Samples were serially diluted and inoculated into C6/36 cells in 96-well plates. After incubation at 28°C for 3 days (CHIKV) or 5 days (DENV), plates were stained using hyper-immune ascetic fluid specific to CHIKV or DENV-1 as primary antibody. A Fluorescein-conjugated goat anti-mouse was used as the second antibody (Biorad).

### Statistical Analysis

Statistical analyses were performed with Stata software (StataCorp LP, Texas, and USA). The numbers of infectious particles in saliva and/or head homogenates were compared using Kruskal-Wallis test. Chi square test was used to compare disseminated infection rate, transmission rate and vector competence for each mosquito/virus pairing.

## Results

### *Ae. albopictus* from France is Susceptible to CHIKV

To compare the susceptibility of *Ae. albopictus* from France (AL-CSM) to CHIKV, *Ae. aegypti* from India (AA-India), where the imported case was originated, was also orally infected with two viral strains: (i) CHIKV 2010–1630 strain that circulated in India in 2010 and (ii) CHIKV 2010–1909 strain isolated from a CHIK autochthonous case from Fréjus. These two viral strains, whose complete genome has been defined, differed only by one single-nucleotide, and showed a 100% identity at the amino acid level. As previously found [2], phylogenetic analysis based on complete genomes confirmed that the two CHIKV strains belong to the ECSA clade, in close relation with strains from India (Figure S1).

To measure the ability of mosquitoes to allow virus to overcome the midgut barrier, disseminated infection rate (DIR) was assessed at days 3, 6, 9 and 14 after the infectious blood-meal. DIR was calculated for each pairing mosquito/virus. After infection with CHIKV, DIR increased along with day pi to reach 100% at day 6 pi (Table 1), illustrating a high susceptibility of both mosquito species to the two CHIKV strains. Slight decreases of DIR were observed from day 6–9 pi to day 14 pi. When examining DIR according to day pi, a significant difference was only found for AA-India infected with CHIKV 2010-1909 (Chi square test: p<0.05). No difference was found according to viral strains and mosquito species (Chi square test: p>0.05).

**Table 1:** Disseminated infection rate, transmission rate and transmission efficiency to CHIKV strains of *Ae. albopictus* from France (AL-CSM) and *Ae. aegypti* from India (AA-INDIA) at different days post-infection.

	AA-India						AL-CSM					
Day pi	CHIKV-1909^†^			CHIKV-1630^‡^			CHIKV-1909^†^			CHIKV-1630^‡^		
	DIR	TR	TE	DIR	TR	TE	DIR	TR	TE	DIR	TR	TE
3	66.7 (15)	50 (10)	33.3 (15)	94.7 (19)	66.7 (18)	63.2 (19)	82.4 (17)	35.7 (14)	29.4 (17)	94.1 (17)	37.5 (16)	35.3 (17)
6	100 (23)	78.3 (23)	78.3 (23)	100 (19)	73.7 (19)	73.7 (19)	100 (19)	84.2 (19)	84.2 (19)	100 (18)	72.2 (18)	72.2 (18)
9	100 (20)	40 (20)	40 (20)	100 (20)	45 (20)	45 (20)	95 (20)	26.3 (19)	25 (20)	94.7 (19)	77.8 (18)	73.7 (19)
14	100 (22)	31.8 (22)	31.8 (22)	85 (20)	47.1 (17)	35 (20)	95.5 (22)	14.3 (21)	13.6 (22)	89.5 (19)	11.8 (17)	10.5 (19)

DIR: Disseminated infection rate corresponding to the proportion of mosquitoes with infected head among tested ones; TR: Transmission rate corresponding to the proportion of mosquitoes with infectious saliva among mosquitoes able to disseminate the virus beyond the midgut barrier; TE: Transmission efficiency corresponding to the proportion of mosquitoes with infectious saliva among tested ones; In parenthesis for DIR, number of mosquitoes analyzed; In parenthesis for TR, number of mosquitoes able to disseminate the virus beyond the midgut; In parenthesis for TE, number of mosquitoes analyzed; ^†^ Strain isolated from a French autochthonous case; ^‡^ Strain isolated from a French imported case.

**Figure 1:**
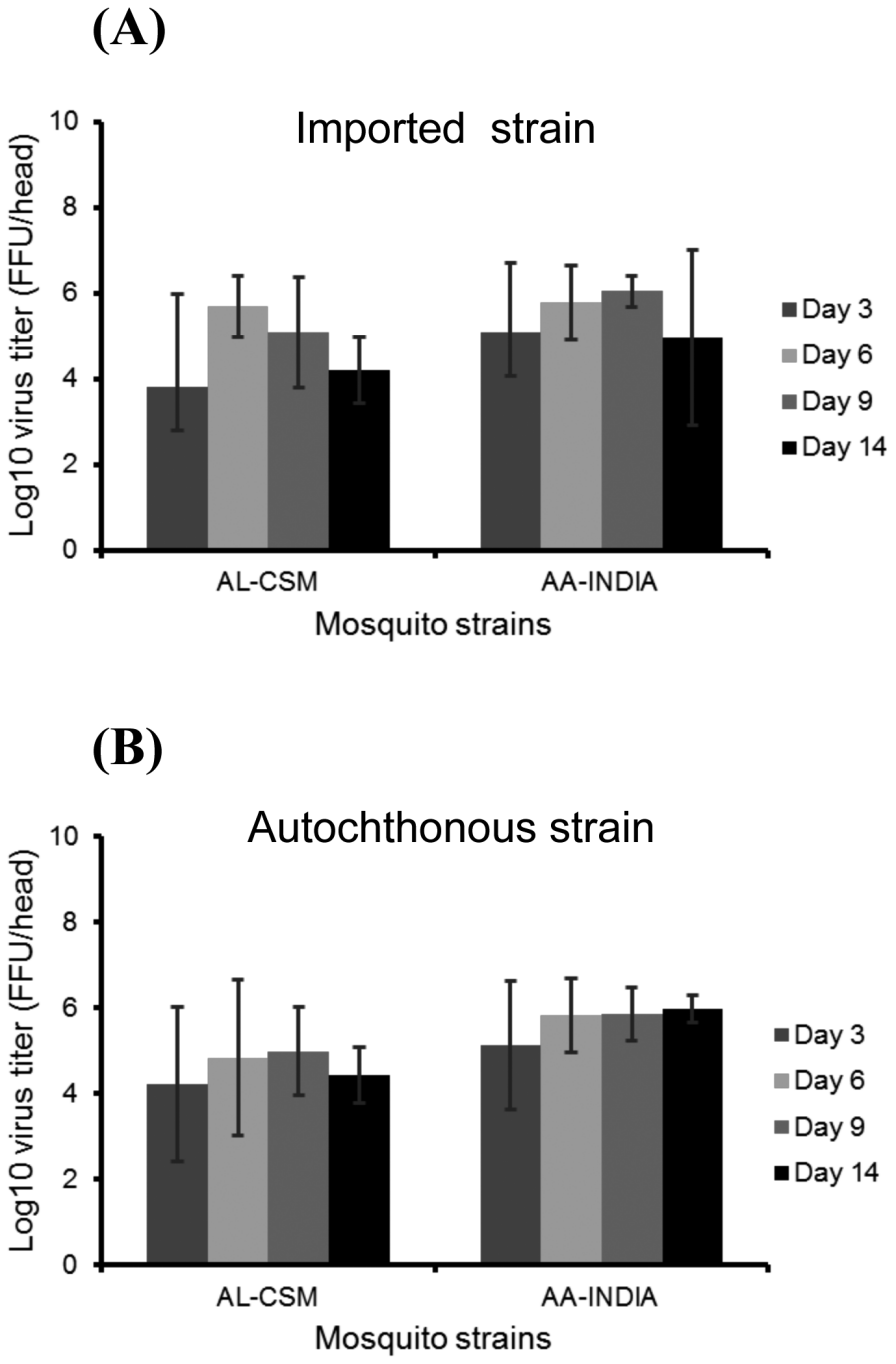
Dissemination of CHIKV in *Ae. albopictus* from France (AL-CSM) and *Ae. aegypti* from India (AA-India). At days 3, 6, 9, and 14 after infection with an infectious blood-meal, mosquitoes were sacrificed and heads were removed for viral titration. The number of infectious particles per head homogenate was estimated by focus fluorescent assay on C6/36 *Ae. albopictus* cells. Two viral strains were tested: (A) CHIKV-1630 strain that circulated in India in 2010 and (B) CHIKV-1909 strain isolated from a CHIKV autochthonous case from Fréjus. Error bars refer to the standard error between different mosquitoes.

The intensity of viral dissemination beyond the midgut barrier can be evaluated by estimating the number of viral particles in head homogenates (Figure 1). For *Ae. albopictus* AL-CSM, the maximum number of CHIKV particles was reached at day 6 pi with 5.7±0.7 log_10_ particles for CHIKV 2010-1630 (Figure 1A) and at day 9 pi with 5.0±1.0 log_10_ particles for CHIKV 2010-1909 (Figure 1B). For *Ae. aegypti* AA-India, the maximum viral load was detected at day 9 pi with 6.1±0.4 log_10_ viral particles for CHIKV 2010-1630 (Figure 1A) and at day 14 pi with 6.0±0.3 log_10_ viral particles for CHIKV 2010-1909 (Figure 1B). When considering each viral strain, AA-India had a slightly higher number of viral particles than AL-CSM at late days pi (Kruskal-Wallis test: p<0.05). When considering each mosquito species, both viral strains were disseminated with similar efficiencies (Kruskal-Wallis test: p>0.05).

To describe the ability of mosquitoes to allow virus to reach the salivary glands after replicating in different mosquito organs and to be transmitted with the saliva delivered by females during blood-feeding, transmission rate (TR) was assessed at days 3, 6, 9 and 14 after the infectious blood-meal. Viral detection in saliva revealed that TR reached a maximum (around 70–80%) at day 6 pi whatever the pairing mosquito/virus, and then declined gradually until day 14 pi (Table 1). When examining mosquito species, a slightly higher proportion of *Ae. aegypti* AA-India was able to transmit CHIKV strains compared to *Ae. albopictus* AL-CSM. No difference was found between the two viral strains (Chi square test: p>0.05) except at day 9 for *Ae. albopictus* AL-CSM (Chi square test: p<0.05). When examining TR according to day pi, no significant difference was found except for *Ae. albopictus* AL-CSM infected with both CHIKV strains (Chi square test: p<0.05).

**Figure 2:**
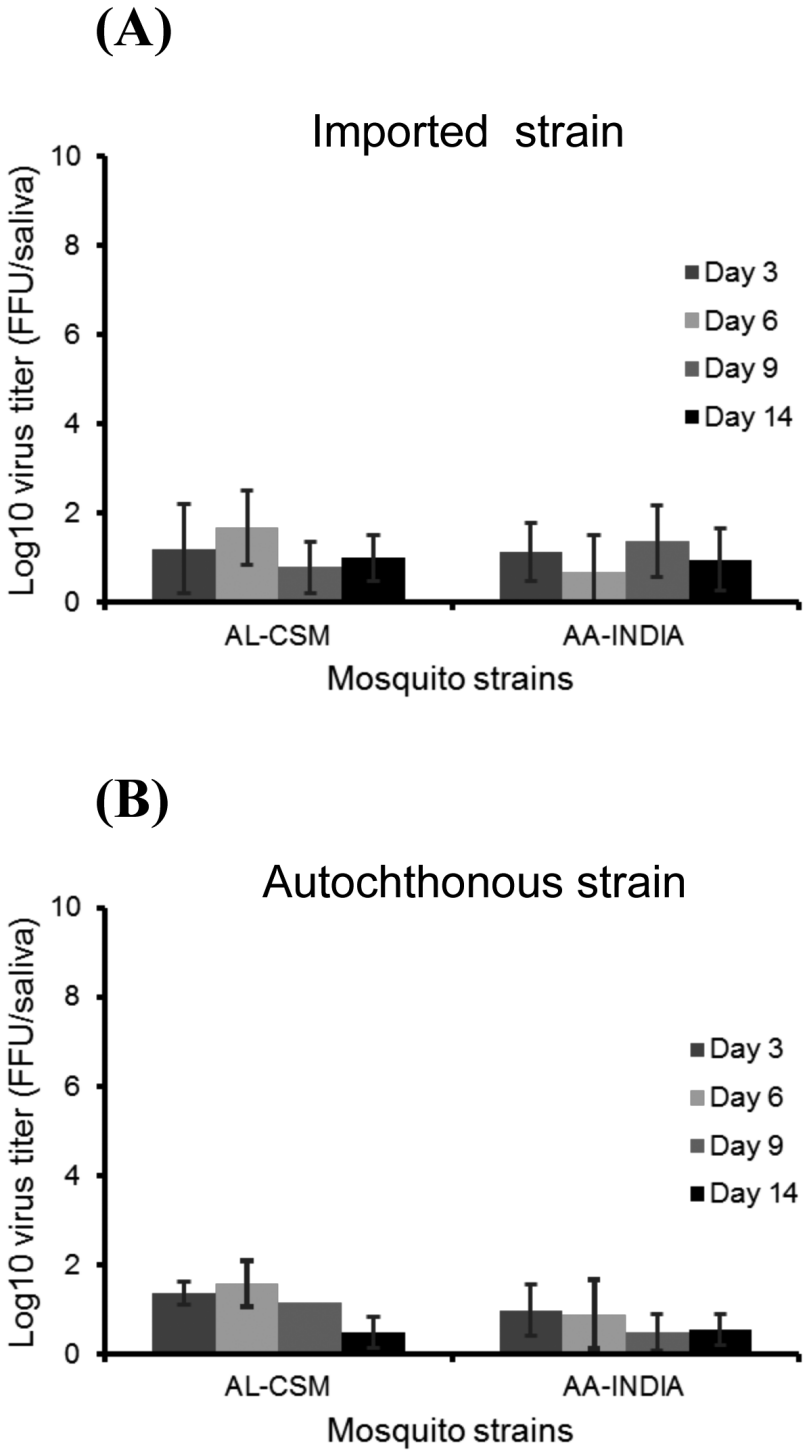
Transmission of CHIKV in saliva of *Ae. albopictus* from France (AL-CSM) and *Ae. aegypti* from India (AA-India). At days 3, 6, 9, and 14 after infection with an infectious blood-meal, mosquitoes were sacrificed and saliva was collected from individual mosquitoes and titrated by focus fluorescent assay on C6/36 *Ae. albopictus* cells. Two viral strains were tested: (A) CHIKV-1630 strain that circulated in India in 2010 and (B) CHIKV-1909 strain isolated from a CHIKV autochthonous case from Fréjus. Error bars refer to the standard error between different mosquitoes.

The intensity of viral transmission can be calculated by estimating the viral load in saliva collected from mosquitoes. As expected, the number of CHIKV particles in saliva was lower than particles estimated in head homogenates (Figure 2). Virus in saliva was detected from day 3 pi and the number of viral particles did not fluctuate significantly along with day pi (Kruskal-Wallis test: p>0.05). For *Ae. albopictus* AL-CSM, the maximum number of CHIKV particles in saliva was reached at day 6 pi with 1.6±0.8 log_10_ particles for CHIKV 2010-1630 (Figure 2A) and 1.6±0.5 log_10_ particles for CHIKV 2010-1909 (Figure 2B). For *Ae. aegypti* AA-India, the maximum viral load in saliva was detected at day 9 pi with 1.5±0.8 log_10_ viral particles for CHIKV 2010-1630 (Figure 2A) and as soon as day 3 pi with 1.1±0.5 log_10_ viral particles for CHIKV 2010-1909 (Figure 2B). Roughly, AL-CSM delivered more viral particles than AA-India for both CHIKV strains (Kruskal-Wallis test: p<0.05).

Ultimately, transmission efficiency (TE) was also defined by estimating the proportion of mosquitoes with virus in saliva among tested ones. When comparing TE between the two viral strains for each mosquito species at a given day pi, no difference was found (Chi square test: p>0.05) except at day 9 pi for *Ae. albopictus* AL-CSM. When examining TE according to day pi for each pairing mosquito/virus, significant difference was found (Chi square test: p<0.05) except for *Ae. aegypti* AA-India infected with the imported CHIKV strain (2010-1630).

These results suggest that a higher proportion of *Ae. aegypti* AA-India was able to transmit both strains of CHIKV but with a lower number of virus in saliva than for *Ae. albopictus* AL-CSM. The imported CHIKV strain (2010–1630) as well as the autochthonous CHIKV strain (2010–1909) behave similarly.

### *Ae. albopictus* from France is Unexpectedly Highly Susceptible to DENV

It is known that *Ae. albopictus* is an unusual vector of DENV compared to the typical vector, *Ae. aegypti.* Therefore, to measure the potential to transmit DENV of *Ae. albopictus* from France (AL-CSM) compared to *Ae. aegypti* from Martinique (AA-Martinique) where the imported case spent some times, mosquitoes were infected with two viral strains: (i) DENV-2025 strain that circulated in Martinique in 2010 and (ii) DENV-1806 strain isolated from a DEN autochthonous case from Nice in 2010. Molecular analysis demonstrated that these two strains belonged to the serotype 1, presenting similarities with strains circulating at the same period in the French West Indies [1]. Complete genome sequence of both DENV strains will be available in a near future (Caro V, personal communication).

Disseminated infection rate (DIR), transmission rate (TR) and transmission efficiency (TE) were assessed at days 9 and 14 after the infectious blood-meal.

Dissemination of DENV in AA-Martinique after ingestion of the infectious blood-meal was characterized by high values of DIR from day 9 pi (∼ >70%) whereas in AL-CSM, DIR fluctuated from 11.5 to 44.8% (Table 2). No difference was detected according to the day pi (Chi square test: p>0.05) except for *Ae. albopictus* AL-CSM infected with the imported DENV strain (2010–2025). Whatever the DENV strain, *Ae. aegypti* AA-Martinique showed higher values of DIR than *Ae. albopictus* AL-CSM (Chi square test: p<0.05) (Table 2). Each mosquito population disseminated equally the two DENV strains (Chi square test: p>0.05).

**Table 2:** Disseminated infection rate, transmission rate and transmission efficiency to DENV strains of *Ae. albopictus* from France (AL-CSM) and *Ae. aegypti* from Martinique (AA-Martinique) at two days post-infection.

	AA-Martinique						AL-CSM						
Day pi	DENV-1806†			DENV-2025‡				DENV-1806†			DENV-2025‡		
	DIR	TR	TE	DIR	TR	TE		DIR	TR	TE	DIR	TR	TE
9	86.4 (22)	21.1 (19)	18.2 (22)	68.4 (19)	15.4 (13)	10.5 (19)		12.5 (48)	66.7 (6)	8.3 (48)	11.5 (52)	66.7 (6)	7.7 (52)
14	96.2 (26)	4 (25)	3.8 (26)	84.2 (19)	12.5 (16)	10.5 (19)		28.1 (32)	55.6 (9)	15.6 (32)	44.8 (29)	61.5 (13)	27.6 (29)

DIR: Disseminated infection rate corresponding to the proportion of mosquitoes with infected head among tested ones; TR: Transmission rate corresponding to the proportion of mosquitoes with infectious saliva among mosquitoes able to disseminate the virus beyond the midgut barrier; TE: Transmission efficiency corresponding to the proportion of mosquitoes with infectious saliva among tested ones; In parenthesis for DIR, number of mosquitoes analyzed; In parenthesis for TR, number of mosquitoes able to disseminate the virus beyond the midgut; In parenthesis for TE, number of mosquitoes analyzed;

† Strain isolated from a French autochthonous case;

‡ Strain isolated from a French imported case.

**Figure 3:**
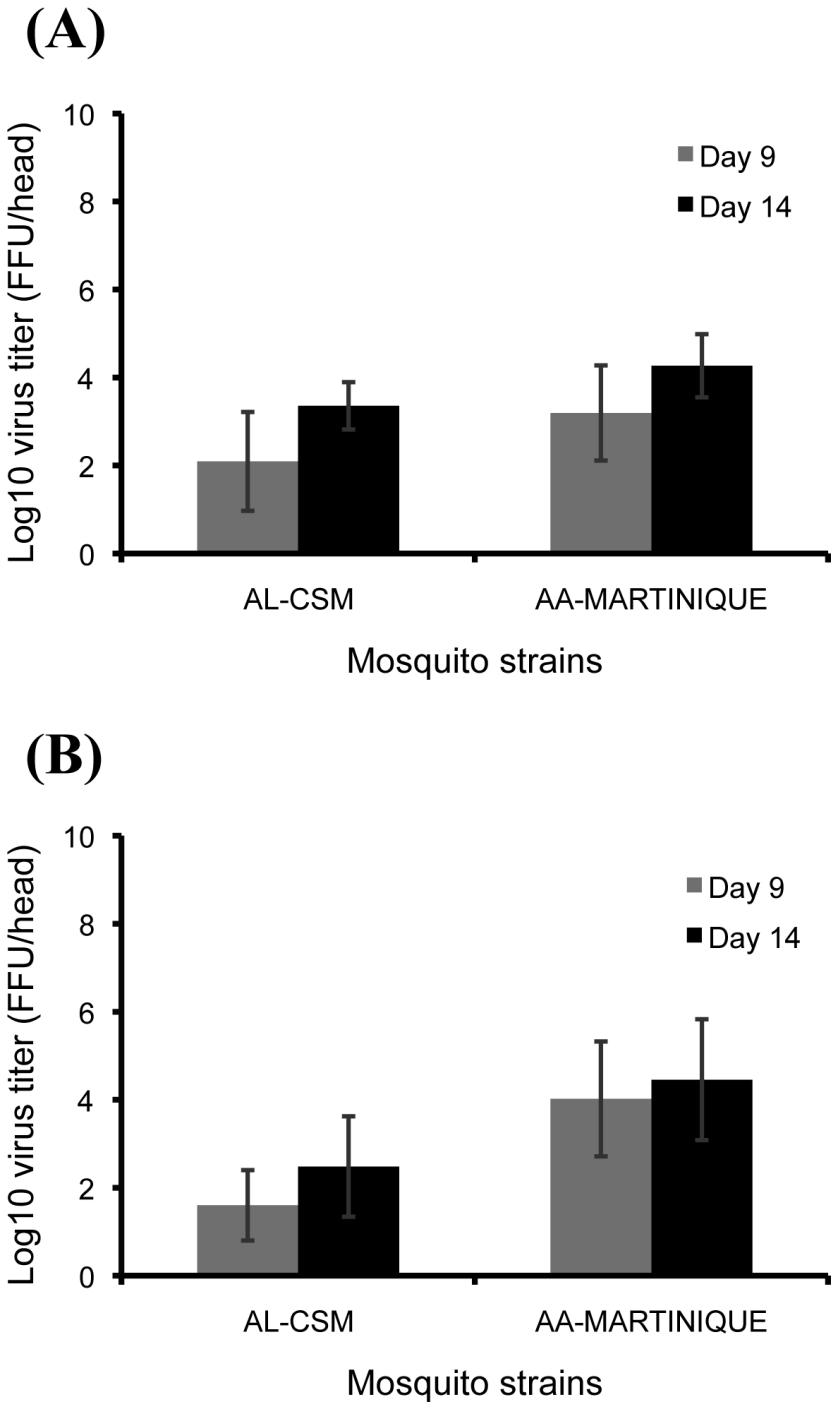
Dissemination of DENV in *Ae. albopictus* from France (AL-CSM) and *Ae. aegypti* from Martinique (AA-Martinique). At days 9 and 14 after infection with an infectious blood-meal, mosquitoes were sacrificed and heads were removed for viral titration. The number of infectious particles per head homogenate was estimated by focus fluorescent assay on C6/36 *Ae. albopictus* cells. Two viral strains were tested: (A) DENV-2025 strain that circulated in Martinique in 2010 and (B) DENV-1806 strain isolated from a DENV-1 autochthonous case from Nice. Error bars refer to the standard error between different mosquitoes.

In order to compare the intensity of viral dissemination, the number of viral particles in head homogenates was estimated. The maximum number of DENV particles was reached at day 14 pi for each pairing mosquito/virus (Figure 3). *Ae. aegypti* AA-Martinique disseminated more DENV particles than *Ae. albopictus* AL-CSM (Kruskal-Wallis test: p<0.05). When comparing the two DENV strains for each mosquito strain, the number of viral particles was roughly similar (Kruskal-Wallis test: p>0.05).

Viral transmission was examined by estimating TR for each pairing mosquito/virus. TRs varied from 4% to 21.4% for *Ae. aegypti* AA-Martinique and from 55.6% to 66.7% for *Ae. albopictus* AL-CSM, with a decrease from day 9 to day 14 pi (Table 2). Higher TRs were obtained for AL-CSM with both DENV strains (Chi square test: p<0.05). No significant differences were found between the two DENV strains for a given mosquito species (Chi square test: p>0.05).

**Figure 4:**
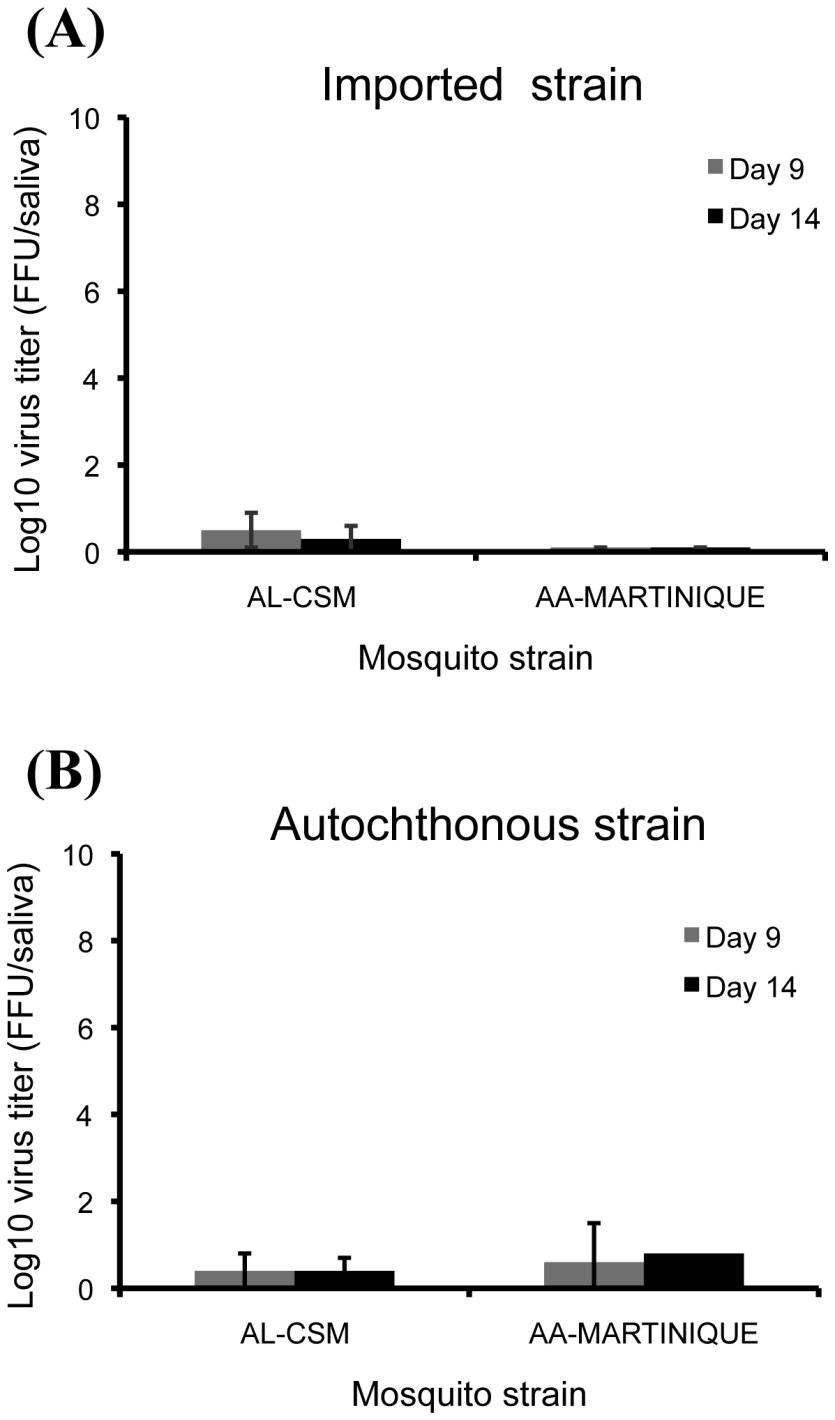
Transmission of DENV in saliva of *Ae. albopictus* from France (AL-CSM) and *Ae. aegypti* from Martinique (AA-Martinique). At days 9 and 14 after infection with an infectious blood-meal provided, mosquitoes were sacrificed and saliva was collected from individual mosquitoes and titrated by focus fluorescent assay on C6/36 *Ae. albopictus* cells. Two viral strains were tested: (A) DENV-2025 strain that circulated in Martinique in 2010 and (B) DENV-1806 strain isolated from a DENV autochthonous case from Nice. Error bars refer to the standard error between different mosquitoes.

As observed with CHIKV, the number of DENV particles in saliva was lower to that obtained in head homogenates (Figure 4). For *Ae. albopictus* AL-CSM, the maximum of DENV particles was reached at day 9 pi with 0.5±0.4 log_10_ particles for DENV 2010-1806 (Figure 4A) and 0.4±0.4 log_10_ particles for DENV 2010–2025 (Figure 4B). For *Ae. aegypti* AA-Martinique, viral loads were also low: 0.1 log_10_ particles for DENV 2010-1806 (Figure 4A) and 0.6±0.9 log_10_ particles for DENV 2010–2025 (Figure 4B) at day 9 pi. AL-CSM and AA-Martinique delivered a number of DENV particles comparable between DENV 2010-1806 and DENV 2010–2025 (Kruskal-Wallis test: p>0.05).

Finally, transmission efficiency (TE) was also defined for each pairing mosquito/virus at different days pi. When examining TE between the two viral strains for each mosquito species at a given day pi, no difference was found (Chi square test: p>0.05). When analyzing TE according to day pi for each pairing mosquito/virus, no significant difference was found (Chi square test: p>0.05) except for *Ae. albopictus* AL-CSM infected with the imported DENV strain (2010–2025) at day 14 pi.

These results suggest that *Ae. albopictus* AL-CSM ensured a low dissemination of DENV even if transmission rate was unexpectedly high. Its TR was high at day 14 pi when infected with the imported DENV strain (2010–2025). Moreover, the typical vector *Ae. aegypti* AA-Martinique achieved a high dissemination of both DENV strains without being correlated with a high transmission. AL-CSM presents the same features for dissemination and transmission of the imported (DENV 2010–2025) as well as the autochthonous (DENV 2010-1806) DENV strains.

## Discussion

Taking advantage of intercontinental trading, *Ae. albopictus*, native to southeast Asia, has extended its geographical range and has brought into light the fear of emerging mosquito borne-diseases. In France, the scare became reality in September 2010, when autochthonous cases of CHIK and DEN were detected in southeast of France, where *Ae. albopictus* has been established since 2004 [18]. Our results show that *Ae. albopictus* from Cagnes-sur-Mer (AL-CSM) was as efficient as the typical tropical vector *Ae. aegypti* to transmit both CHIKV and DENV.

Populations of *Ae. albopictus* are currently established on the eastern Mediterranean coastline of France. Its capacity to lay cold-resistant eggs has facilitated the colonization of new geographic areas in northern latitudes (reviewed in [3]; [19]). In addition, its preference for small artificial containers as breeding sites in domestic environments has increased potential close contacts with humans (reviewed in [7]). *Ae. albopictus* can reach high densities in August-September as climatic conditions are favorable [18]. In addition, travelers infected with CHIKV and DENV returning from countries where these arboviruses are endemic or where epidemics are taking place have been frequently reported in Europe, including France [1], [20]. These conditions together are essential for the transmission of imported arboviruses to take place. Our laboratory viral challenges were implemented in conditions allowing optimal viral replication in tropical mosquito species: an incubation temperature of 28°C and an incubation period (day 3 to day 14 for CHIKV and day 9 to day 14 pi for DENV) suitable for CHIKV [17] and DENV infecting *Ae. aegypti* [21]. Experimental infections were performed with blood-meals providing viral titers compatible with viremia levels recorded in patients [22], [23]. Temperate populations such as AL-CSM are expected to be disadvantaged in such conditions, but our results were not consistent with these expectations. Several environmental factors such as temperature may affect vector capacity by modifying notably vector dynamics [24] and vector competence [25]. For example, recent studies have shown that the incubation temperature of immature stages alters susceptibility of *Ae. albopictus* for CHIKV [26]. Although *Ae. albopictus* was already shown to be susceptible to both viruses in the laboratory, its high vector competence in optimal conditions compared to the typical vector was quite surprising.

The analyzed CHIKV strain isolated in Fréjus from an imported human case belongs to the ECSA clade and harbors an alanine at the position E1-226. Based on our laboratory viral challenges, we found that both CHIKV strains (autochthonous and imported) were efficiently transmitted by *Ae. albopictus* AL-CSM and *Ae. aegypti* AA-India with around 35–67% of mosquitoes able to ensure viral dissemination delivering up to 14 viral particles at day 3 pi. Using a similar titer of blood-meal (i.e., 10^7.5 ^PFU/mL), we showed in a previous work that *Ae. albopictus* from La Reunion Island was very efficient to transmit CHIKV E1-226V delivering around 11 viral particles at day 3 pi [17]. The substitution E1-226A by E1-226V has been demonstrated to be responsible of the improved transmission in *Ae. albopictus* [27], [28]. Nevertheless, the pairing *Ae. albopictus* AL-CSM and CHIKV E1-226A was found to be unexpectedly efficient. *Ae. albopictus* AL-CSM is a temperate species experiencing low temperatures which can express a higher competence to vector arboviruses [26]. Another substitution at position E2-211 with a threonine instead of an isoleucine may increase the potential of CHIKV transmission in *Ae. albopictus* when E1-226V is also present. The mutation E2-I211T significantly increases infectivity of CHIKV for *Ae. albopictus* when expressed together with the E1-226V mutation [29]. It was suggested that this region of the E2 protein constitutes a cell-receptor binding domain. Mutations might affect interactions of the virus with putative receptor(s) present on midgut cells [30]. The detection of E2-211T in CHIKV isolates from France can favor the E1-226V genotype to reach fixation after several rounds of autochthonous transmission cycles involving *Ae. albopictus*. The continuous circulation of CHIKV since its emergence in 2005 has led to the selection of other mutations enhancing the effect of E1-226V for transmission in *Ae. albopictus* [31]. In India, circulating CHIKV genotypes belong to the newly introduced ECSA lineage [2]. Two specific mutations, E1-K211E and E2-V264A, were detected in CHIKV strains isolated from New-Delhi revealing the emergence of a new signature in this clade [32]. Thus, other positions in the CHIKV genome exert strong epistatic effects on the E1-A226V substitution responsible for a significant increase in CHIKV transmission by *Ae. albopictus*. These effects are lineage-specific [29], [31].

The Dengue 1 strain, isolated from a patient in Nice, originated from Martinique in the French West Indies where an on-going epidemic took place in 2010. The mosquito *Ae. aegypti* is the main vector involved in most urban dengue outbreaks worldwide [33]. However, *Ae. albopictus* may act as a secondary vector [34] although in a recent past, it was implicated in outbreaks in Hawaii in 2001–2002 [35] and La Réunion Island in 2004 [36]. *Ae. aegypti* populations from Martinique are highly competent to DENV [37]. Nevertheless, *Ae. albopictus* from Cagnes-sur-Mer was found to be efficient in transmitting both strains of DENV-1 (autochthonous and imported) isolated from patients in Nice. Almost 67% of *Ae. albopictus* AL-CSM which have ensured viral dissemination were able to transmit DENV at day 9 after the ingestion of the infectious blood-meal. On the other side, only 21% of *Ae. aegypti* AA-Martinique were able to deliver DENV. The high efficiency of *Ae. albopictus* from Cagnes-sur-Mer to transmit DENV-1 is really unusual and contrasts with its counterpart *Ae. albopictus* from another Mediterranean country, Lebanon; it was capable to transmit only at day 21 pi with 38% of mosquitoes delivering 174±455 viral particles [38].

For both viruses, viral dissemination from the midgut to colonize organs bathed in the hemolymph was more efficient for the typical vector, *Ae. aegypti*. Later, release of viral particles from the salivary glands through saliva expectorated by the mosquito was favored in *Ae. albopictus*. We hypothesized that viral replication in midgut cells is essential to acquire enough infectivity for the virus to be transmitted in an optimal way. A direct competition of assays *in vivo* between the E1-226V and E1-226A variants highlighted the selective role of the midgut barrier. Indeed, when intrathoracically inoculated into the mosquito hemocele, CHIKV bypasses the midgut cells and infect directly secondary organs such as the salivary glands without any selection operating; the transmission of the E1-226V variant by *Ae. albopictus* is no longer favored (Arias-Goeta et al., unpublished data). Thus, this mutation mainly occurs before the virus is released from midgut cells into hemocele. The midgut plays a key role in enhanced transmission of E1-226V in *Ae. albopictus* as suggested by previous findings [27], [28]. The E1-226A seems to adopt the same features in *Ae. albopictus* AL-CSM. Similar studies should be initiated with DENV. In addition, for each virus, genomes of both strains (autochthonous and imported) are quite similar, suggesting that more autochthonous transmission cycles involving native *Ae. albopictus* would be necessary to generate more adapted viruses for an enhanced transmission by this vector. Moreover, mosquitoes are persistently infected and the mosquito innate immune responses play a key role in controlling infection [39]. This would contribute to explain decrease in dissemination and transmission efficiencies at late days post-infection.

Autochthonous transmission of CHIK and DEN in continental Europe is thus possible. While occurrence of CHIK outbreaks has been proven possible in the past [40], the establishment of DEN transmission in southeastern France or further spread in Europe seems to be unexpected. Nevertheless, the high efficiency of *Ae. albopictus* from Cagnes-sur-Mer to transmit both viruses in the laboratory leads us to avoid underestimating *Ae. albopictus* from temperate regions to sustain epidemic outbreaks. Environmental conditions prevailing in the southeast of France are suitable enough to sustain outbreaks during favorable seasons, as evidenced by the Italian outbreak of CHIKV occurring in the province of Ravenna at the same latitude than the French cities, Nice and Fréjus.

## Supporting Information

Figure S1 **Phylogenetic relationships among several CHIKV and ones from French cases (Genbank accession number pending) based on complete genome (11,237 nucleotides) analysis.** Bootstrap support values (1,000 replicates) are indicated at major nodes. Gray shading indicates the two French CHIKV isolates. Scale bar indicates number of base substitutions per site. ECSA, East/Central/South Africa.
(PDF)

## References

[1] La Ruche G, Souares Y, Armengaud A, Peloux-Petiot F, Delaunay P, et al (2010) First two autochthonous dengue virus infections in metropolitan France, September 2010. Euro Surveill 15: 19676. 20929659

[2] Grandadam M, Caro V, Plumet S, Thiberge JM, Souares Y, et al (2011) Chikungunya virus, southeastern France. Emerg Infect Dis 17: 910–913. 21529410 10.3201/eid1705.101873 PMC3321794

[3] Medlock JM, Hansford KM, Schaffner F, Versteirt V, Hendrickx G, et al (2012) A review of the invasive mosquitoes in Europe: ecology, public health risks, and control options. Vector Borne Zoonotic Dis 12: 435–447. 22448724 10.1089/vbz.2011.0814 PMC3366101

[4] Adhami J, Reiter P (1998) Introduction and establishment of *Aedes* (*Stegomyia*) *albopictus* Skuse (Diptera: Culicidae) in Albania. J Am Mosq Control Assoc 14(3): 340–343. 9813831

[5] Delaunay P, Jeannin C, Schaffner F, Marty P (2009) News on the presence of the tiger mosquito *Aedes albopictus* in metropolitan France. Arch Pediatr 16 Suppl 2S66–71. 19836679 10.1016/S0929-693X(09)75304-7

[6] Scholte EJ, Schaffner F (2007) Waiting for the tiger: establishment and spread of the Asian tiger mosquito in Europe. In: Takken W, Knols B, eds. Emerging pests and vector-borne diseases in Europe, Wageningen Academic Publishers, Wageningen, The Netherlands. 241–260.

[7] Paupy C, Delatte H, Bagny L, Corbel V, Fontenille D (2009) *Aedes albopictus*, an arbovirus vector: from the darkness to the light. Microbes Infect 11: 1177–1185. 19450706 10.1016/j.micinf.2009.05.005

[8] Moutailler S, Barre H, Vazeille M, Failloux AB (2009) Recently introduced *Aedes albopictus* in Corsica is competent to Chikungunya virus and in a lesser extent to dengue virus. Trop Med Int Health 14: 1105–1109. 19725926 10.1111/j.1365-3156.2009.02320.x

[9] Talbalaghi A, Moutailler S, Vazeille M, Failloux AB (2010) Are *Aedes albopictus* or other mosquito species from northern Italy competent to sustain new arboviral outbreaks? Med Vet Entomol 24: 83–87. 20377735 10.1111/j.1365-2915.2009.00853.x

[10] Rezza G, Nicoletti L, Angelini R, Romi R, Finarelli AC, et al (2007) Infection with chikungunya virus in Italy: an outbreak in a temperate region. Lancet 370: 1840–1846. 18061059 10.1016/S0140-6736(07)61779-6

[11] Gould EA, Gallian P, De Lamballerie X, Charrel RN (2010) First cases of autochthonous dengue fever and chikungunya fever in France: from bad dream to reality! Clin Microbiol Infect. 16: 1702–1704. 10.1111/j.1469-0691.2010.03386.x 21040155

[12] Schmidt-Chanasit J, Haditsch M, Schoneberg I, Gunther S, Stark K, et al.. (2010) Dengue virus infection in a traveler returning from Croatia to Germany. Euro Surveill 15. 10.2807/ese.15.40.19677-en 20946759

[13] Gjenero-Margan I, Aleraj B, Krajcar D, Lesnikar V, Klobucar A, et al.. (2011) Autochthonous dengue fever in Croatia, August-September 2010. Euro Surveill 16. 21392489

[14] Receveur M, Ezzedine K, Pistone T, Malvy D (2010) Chikungunya infection in a French traveller returning from the Maldives, October, 2009. Euro Surveill 15: 19494. 20197023 10.2807/ese.15.08.19494-en

[15] Schuffenecker I, Iteman I, Michault A, Murri S, Frangeul L, et al (2006) Genome microevolution of chikungunya viruses causing the Indian Ocean outbreak. PLoS Med 3: e263. 16700631 10.1371/journal.pmed.0030263 PMC1463904

[16] Mousson L, Zouache K, Arias-Goeta C, Raquin V, Mavingui P, et al (2012) The native Wolbachia symbionts limit transmission of dengue virus in *Aedes albopictus* . PLoS Negl Trop Dis 6: e1989. 23301109 10.1371/journal.pntd.0001989 PMC3531523

[17] Dubrulle M, Mousson L, Moutailler S, Vazeille M, Failloux AB (2009) Chikungunya virus and *Aedes* mosquitoes: saliva is infectious as soon as two days after oral infection. PLoS One 4: e5895. 19521520 10.1371/journal.pone.0005895 PMC2690823

[18] Delaunay P, Mathieu B, Marty P, Fauran P, Schaffner F (2007) Chronology of the development of *Aedes albopictus* in the Alpes-Maritimes Department of France, from 2002 to 2005. Med Trop 67: 310–311. 17784689

[19] Thomas SM, Obermayr U, Fischer D, Kreyling J, Beierkuhnlein C (2012) Low-temperature threshold for egg survival of a post-diapause and non-diapause European aedine strain, *Aedes albopictus* (Diptera: Culicidae). Parasit Vectors 5: 100. 22621367 10.1186/1756-3305-5-100 PMC3403971

[20] Odolini S, Parola P, Gkrania-Klotsa E, Caumes E, Schlagenhauf P, et al (2012) Travel-related imported infections in Europe, EuroTravNet 2009. Clin Microbiol Infec 18: 468–474. 21848975 10.1111/j.1469-0691.2011.03596.x

[21] Salazar MI, Richardson JH, Sanchez-Vargas I, Olson KE, Beaty BJ (2007) Dengue virus type 2: replication and tropisms in orally infected *Aedes aegypti* mosquitoes. BMC Microbiol 7: 9. 17263893 10.1186/1471-2180-7-9 PMC1797809

[22] Parola P, de Lamballerie X, Jourdan J, Rovery C, Vaillant V, et al (2006) Novel chikungunya virus variant in travelers returning from Indian Ocean islands. Emerg Infect Dis 12: 1493–1499. 17176562 10.3201/eid1210.060610 PMC3290960

[23] Wang WK, Chen HL, Yang CF, Hsieh SC, Juan CC, et al (2006) Slower rates of clearance of viral load and virus-containing immune complexes in patients with dengue hemorrhagic fever. Clin Infect Dis 43: 1023–1030. 16983615 10.1086/507635

[24] Alto BW, Juliano SA (2001) Temperature effects on the dynamics of *Aedes albopictus* (Diptera: Culicidae) populations in the laboratory. J Med Entomol 38: 548–556. 11476335 10.1603/0022-2585-38.4.548 PMC2579928

[25] Muturi EJ, Alto BW (2011) Larval environmental temperature and insecticide exposure alter *Aedes aegypti* competence for arboviruses. Vector Borne Zoonotic Dis 11: 1157–1163. 21453010 10.1089/vbz.2010.0209

[26] Westbrook CJ, Reiskind MH, Pesko KN, Greene KE, Lounibos LP (2010) Larval Environmental Temperature and the Susceptibility of *Aedes albopictus* Skuse (Diptera: Culicidae) to Chikungunya Virus. Vector Borne Zoonotic Dis 10: 241–247. 19725768 10.1089/vbz.2009.0035 PMC2883477

[27] Vazeille M, Moutailler S, Coudrier D, Rousseaux C, Khun H, et al (2007) Two Chikungunya isolates from the outbreak of La Reunion (Indian Ocean) exhibit different patterns of infection in the mosquito, *Aedes albopictus* . PLoS One 2: e1168. 18000540 10.1371/journal.pone.0001168 PMC2064959

[28] Tsetsarkin KA, Vanlandingham DL, McGee CE, Higgs S (2007) A single mutation in chikungunya virus affects vector specificity and epidemic potential. PLoS Pathog 3: e201. 18069894 10.1371/journal.ppat.0030201 PMC2134949

[29] Tsetsarkin KA, McGee CE, Volk SM, Vanlandingham DL, Weaver SC, et al (2009) Epistatic roles of E2 glycoprotein mutations in adaption of chikungunya virus to *Aedes albopictus* and *Ae. aegypti* mosquitoes. PLoS One 4: e6835. 19718263 10.1371/journal.pone.0006835 PMC2729410

[30] Myles KM, Pierro DJ, Olson KE (2003) Deletions in the putative cell receptor-binding domain of Sindbis virus strain MRE16 E2 glycoprotein reduce midgut infectivity in *Aedes aegypti* . J Virol 77: 8872–8881. 12885905 10.1128/JVI.77.16.8872-8881.2003 PMC167217

[31] Tsetsarkin KA, Chen R, Leal G, Forrester N, Higgs S, et al (2011) Chikungunya virus emergence is constrained in Asia by lineage-specific adaptive landscapes. Proc Natl Acad Sci U S A 108: 7872–7877. 21518887 10.1073/pnas.1018344108 PMC3093459

[32] Shrinet J, Jain S, Sharma A, Singh SS, Mathur K, et al (2012) Genetic characterization of Chikungunya virus from New Delhi reveals emergence of a new molecular signature in Indian isolates. Virol J 9: 100. 22632412 10.1186/1743-422X-9-100 PMC3495852

[33] Jansen CC, Beebe NW (2010) The dengue vector *Aedes aegypti*: what comes next. Microbes Infect 12: 272–279. 20096802 10.1016/j.micinf.2009.12.011

[34] Lambrechts L, Scott TW, Gubler DJ (2010) Consequences of the expanding global distribution of *Aedes albopictus* for dengue virus transmission. PLoS Negl Trop Dis 4: e646. 20520794 10.1371/journal.pntd.0000646 PMC2876112

[35] Effler PV, Pang L, Kitsutani P, Vorndam V, Nakata M, et al (2005) Dengue fever, Hawaii, 2001–2002. Emerg Infect Dis 11: 742–749. 15890132 10.3201/eid1105.041063 PMC3320380

[36] Pierre V, Thiria J, Rachou E, Sissoko D, Lassale C, et al. (2005) Epidémie de dengue 1 à la Réunion en 2004. Journées INVS, 2005. Available: http://www.invs.sante.fr/publications/2005/jvs_2005/poster_13.pdf.

[37] Yebakima A, Charles C, Mousson L, Vazeille M, Yp-Tcha MM, et al (2004) Genetic heterogeneity of the dengue vector *Aedes aegypti* in Martinique. Trop Med Int Health 9: 582–587. 15117302 10.1111/j.1365-3156.2004.01241.x

[38] Haddad N, Mousson L, Vazeille M, Chamat S, Tayeh J, et al (2012) *Aedes albopictus* in Lebanon, a potential risk of arboviruses outbreak. BMC Infect Dis 12: 300. 23151056 10.1186/1471-2334-12-300 PMC3519687

[39] Fragkoudis R, Attarzadeh-Yazdi G, Nash AA, Fazakerley JK, Kohl A (2009) Advances in dissecting mosquito innate immune responses to arbovirus infection. J Gen Virol 90: 2061–2072. 19570957 10.1099/vir.0.013201-0

[40] Rezza G (2008) Re-emergence of Chikungunya and other scourges: the role of globalization and climate change. Ann Ist Super Sanita 44: 315–318. 19351987

