# Supplementary material for: High Efficiency of Temperate Aedes albopictus to Transmit Chikungunya and Dengue Viruses in the Southeast of France
Source: PLoS One. 2013 Mar 18;8(3):e59716. doi: 10.1371/journal.pone.0059716 (PMC3601061; doi:10.1371/journal.pone.0059716)

Figure S1. Phylogenetic relationships among several CHIKV and ones from French cases (Genbank accession number pending) based on complete genome (11,237 nucleotides) analysis. Bootstrap support values (1,000 replicates) are indicated at major nodes. Gray shading indicates the two French CHIKV isolates. Scale bar indicates number of base substitutions per site. ECSA, East/Central/South Africa.

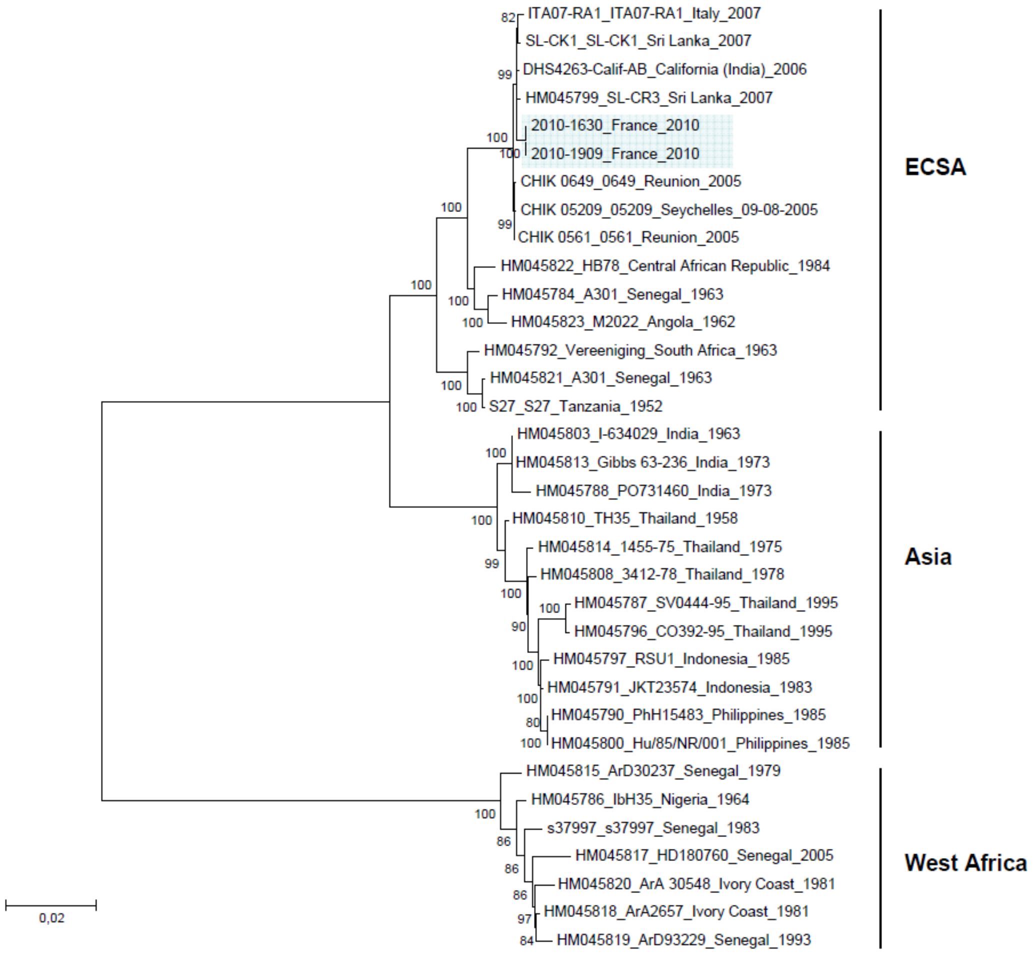

Supplement: Figure S1 — Phylogenetic relationships among several CHIKV and ones from French cases (Genbank accession number pending) based on complete genome (11,237 nucleotides) analysis. Bootstrap support values (1,000 replicates) are indicated at major nodes. Gray shading indicates the two French CHIKV isolates. Scale bar indicates number of base substitutions per site. ECSA, East/Central/South Africa. (PDF) [file pone.0059716.s001.pdf]
